# Virion Assembly Factories in the Nucleus of Polyomavirus-Infected Cells

**DOI:** 10.1371/journal.ppat.1002630

**Published:** 2012-04-05

**Authors:** Kimberly D. Erickson, Cedric Bouchet-Marquis, Katie Heiser, Eva Szomolanyi-Tsuda, Rabinarayan Mishra, Benjamin Lamothe, Andreas Hoenger, Robert L. Garcea

**Affiliations:** 1 Department of Molecular, Cellular and Developmental Biology, University of Colorado, Boulder, Colorado, United States of America; 2 The Biofrontiers Institute, University of Colorado, Boulder, Colorado, United States of America; 3 Department of Pathology, University of Massachusetts Medical School, Worcester, Massachusetts, United States of America; Brown University, United States of America

## Abstract

Most DNA viruses replicate in the cell nucleus, although the specific sites of virion assembly are as yet poorly defined. Electron microscopy on freeze-substituted, plastic-embedded sections of murine polyomavirus (PyV)-infected 3T3 mouse fibroblasts or mouse embryonic fibroblasts (MEFs) revealed tubular structures in the nucleus adjacent to clusters of assembled virions, with virions apparently “shed” or “budding” from their ends. Promyelocytic leukemia nuclear bodies (PML-NBs) have been suggested as possible sites for viral replication of polyomaviruses (BKV and SV40), herpes simplex virus (HSV), and adenovirus (Ad). Immunohistochemistry and FISH demonstrated co-localization of the viral T-antigen (Tag), PyV DNA, and the host DNA repair protein MRE11, adjacent to the PML-NBs. In PML**^−/−^** MEFs the co-localization of MRE11, Tag, and PyV DNA remained unchanged, suggesting that the PML protein itself was not responsible for their association. Furthermore, PyV-infected PML**^−/−^** MEFs and PML**^−/−^** mice replicated wild-type levels of infectious virus. Therefore, although the PML protein may identify sites of PyV replication, neither the observed “virus factories” nor virus assembly were dependent on PML. The ultrastructure of the tubes suggests a new model for the encapsidation of small DNA viruses.

## Author Summary

Polyomaviruses are infectious pathogens of mammals and birds that have been linked to the development of cancers in their hosts. Members of the polyomavirus family are associated with human disease, such as JCV and BKV, and over the past few years, several more human polyomaviruses (WUV, KIV and MCV) have been discovered in immune-suppressed individuals. We are studying the way in which these viruses assemble in cells in order to identify critical points where anti-viral therapies could target these viruses. Using a structural, biochemical and cell biological approach, we set out to define sites of virus assembly and virus intermediates. We identified virus-specific structures that we termed “virus factories”. We believe that these sites serve as an assembly line for the production of new viruses. Our study provides new evidence for the presence and composition of virus assembly factories, and identifies a host protein that may be important for infection by polyomaviruses.

## Introduction

Increasing evidence suggests that the assembly of many viruses occurs at specific intracellular sites, which have been termed “virus factories” [1], [2]. These subcellular domains are gathering points for coordinating genome replication and capsid protein assembly into virions. The ultrastructure of the factories has been determined for a number of RNA viruses that assemble in the cytoplasm. For example, specific membrane compartments such as the ER appear to be co-opted and re-configured such that the surface of the membrane is used as a scaffold, where viral replication is spatially juxtaposed with capsid proteins specifically delivered to these locations [3]–[5]. Such scaffolds likely have a significant kinetic impact on virion assembly. Structural information concerning assembly sites for DNA viruses that replicate and assemble in the nucleus is less clear, perhaps due to the intrinsic complexity and dynamic nature of the nuclear architecture.

Polyomaviruses are small non-enveloped dsDNA viruses that replicate and assemble in the cell nucleus. The viral capsid is comprised of 72 pentamers of the major capsid protein VP1, each associated with a single copy of a minor capsid protein, either VP2 or VP3 [6]. A complex of VP1 pentamer, with VP2/3, is nuclear imported as a capsid “subunit” late in infection [7]. Replication of the viral genome is accomplished through the interaction of the viral large T-antigen (Tag) and its recruitment of cellular DNA replication proteins [8], [9]. How the capsid protein subunits specifically identify the viral genome is unknown, but sequences near the viral origin of replication along with the J-domain of large Tag both appear to have important functions [10]–[13]. In contrast to the larger DNA viruses, such as herpes [14] and Ad [15], assembly of polyomavirus has been hypothesized to involve “polymerization” of the capsid protein subunits onto the viral minichromosomes rather than injection of the viral genome into a preformed capsid structure [16]. However, intermediates in the assembly process are not well defined.

Candidate sites for nuclear virus assembly factories are promyelocytic leukemia nuclear bodies or PML-NBs (previously termed ND10s, PML oncogenic domains, or PODs). Maul *et al* first associated PML-NBs with DNA virus replication, specifically studying infection by herpes simplex virus type-1 (HSV-1), adenovirus type 5 (Ad5), and SV40 [17]–[20]. PML-NBs are functionally heterogeneous intra-nuclear structures, operationally identified by immuno-staining with anti-PML protein antibodies (*i.e.*, a subset of nuclear “dots”). They have been associated with such diverse functions as interferon antiviral responses, DNA damage repair, and the p53 response [21], [22]. Although a large number of proteins have been found associated with PML-NBs, constitutive proteins include PML, which appears essential for PML-NB component protein co-localization, along with Sp100 and Daxx. PML-NB formation requires PML modification by sumoylation [23]. Using 4Pi fluorescence laser-scanning microscopy, PML-NBs have been modeled in three dimensions as spheres of varying diameters with a 50–100 nm thick shell comprised of PML and Sp100 proteins stabilized by sumoylation [24]. PML-NBs in uninfected cells may serve as scaffolds for assembling a variety of DNA repair and checkpoint signaling proteins in response to DNA damage, such as that caused by UV-irradiation [25], [26]. Many DNA viruses appear to usurp the cellular DNA repair proteins for their replication [27], and if replication and virion assembly are spatially coupled, then perhaps these viruses also utilize PML-NBs for capsid assembly.

Polyomavirus (SV40, BKV, JCV) DNA replication has been localized adjacent to PML-NBs [17], [28]–[30], and associated with recruitment of cellular DNA damage-related proteins, such as ATM kinase and the MRN complex [31], [32]. In contrast to the large DNA viruses, infection with polyomaviruses does not grossly disrupt the morphology of PML-NBs, although their number and size may increase and alterations in their function may be detected [30]. Tag and VP1 have been co-localized with PML-NBs during BKV, SV40 and JCV infection supporting a spatial coupling of replication with assembly [28], [29]. PML-NBs also are associated with papillomavirus (PV) replication [33]–[38]. Day *et al* have suggested a model in which the L2 protein first localizes to PML-NBs, associates with the viral E2 protein, which specifically labels the PV viral genome, and subsequently recruits the major capsid protein L1 to the genome for virion assembly [33], [38]. However, neither E2-dependent transcription nor PV DNA replication have been found to be dependent on PML-NBs, and the PV L2 capsid protein does not require the PML protein itself for localization into specific foci or to form virus-like particles [37], [39].

The convergence of Tag, DNA repair proteins, replicating viral DNA, and capsid proteins at PML-NBs during polyomavirus infection suggests that these sites may function as virus factories where all components in the assembly process interact. We have used electron microscopy and tomography of cryo-preserved samples to identify ultrastructural elements that might represent these factories in mouse polyomavirus (PyV)-infected mouse cells. Adjacent to growing virion clusters we identified tubular structures that appear to give rise to new virions. These structures were independent of the presence of the PML protein, and PML^−/−^ cells and knockout mice were unaffected in virus replication. Moreover, the ultrastructure of the tubules suggests a new model for small DNA virus encapsidation.

## Results

### Temporal Progression of Nuclear Virus Assembly

**Figure 1:**
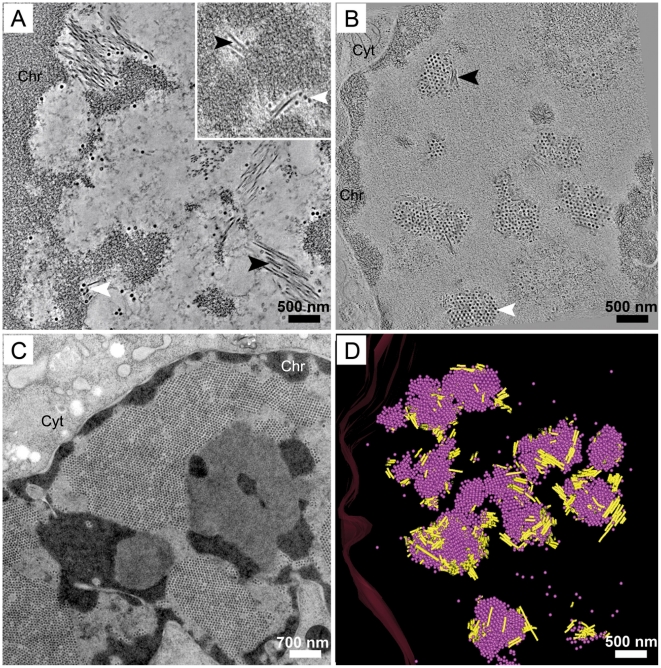
Temporal progression of PyV nuclear assembly. Images from dual-axis tomograms of high pressure frozen, Epon-embedded, 300 nm thick sections of 3T3 cells infected with PyV (MOI of 10–20 pfu/cell) and harvested at 32 hpi. **A**) Tubular structures (black arrowhead) are present in the periphery of the condensed chromatin (Chr) adjacent to occasional virions (white arrowhead). **B**) A 1 nm section extracted from a 2×2 montage over six serial sections (1.8 µm thick) of a 3T3 nucleus in which the interchromatin space is partially filled with virion clusters and each cluster is associated with tubular structures. As infection proceeds, the number of virus clusters (white arrowhead) increases while the tubular structures (black arrowhead) are less prominent. **C**) Late in infection virions fill the entire interchromatin space and tubular structures are not seen. **D**) 3-D model of the 2×2 montage over six serial sections (each 300 nm thick) of a PyV-infected 3T3 nucleus. The model represents 1.8 µm thick section of the nucleus showing the connections between virion clusters and tubular structures. An image extracted from the tomogram is shown in (B); a video of the tomograms of each section and the model can be found as supporting information Video S1. Pink spheres, full virions; yellow cylinders, tubular structures. Chr, host condensed chromatin; Cyt, cytoplasm.

We used electron microscopy and tomographic three-dimensional (3-D) reconstructions of freeze-substituted, plastic-embedded specimens to identify possible sites and structural features of PyV virion assembly. PyV-infected mouse 3T3 fibroblasts were preserved by high-pressure freezing followed by low temperature freeze-substitution and resin embedding. PyV infection proceeds asynchronously and rapidly, consequently progressive stages of infection stages are observed in a single sample. Thus, the time course of infection was operationally-defined based upon the presence, size and number of virus clusters observed in the nucleus of an individual cell. Early in infection full virions were somewhat dispersed throughout the volume of the nucleus and not yet arranged in clusters, although they were closely associated with tubular structures (Figure 1A and inset). The tubular structures were often located near the periphery of cellular condensed chromatin (Figure 1A), consistent with previous observations for JCV [29], [40]. As infection proceeded, the number of virions increased, forming virus clusters or arrays, remaining associated with the tubes (Figure 1B, data not shown). The tubular structures were absent, or could not be detected, late in infection when arrays of progeny virions filled the nucleus (Figure 1C). The temporal appearance of the tubes is consistent with their function as an assembly intermediate.

When a montage of tomograms of Epon-embedded, semi-thick (300 nm) serial sections from a PyV-infected 3T3 cell (Figure 1B) was modeled, the seemingly isolated virus clusters were actually interconnected by the tubular structures (Figure 1D; see Supporting information Video S1). Additionally, the sites of virion accumulation were no longer restricted to the periphery of the nucleus, but rather virus clusters and tubes were found throughout the interchromatin space (Figure 1B). The tubular structures were not observed in uninfected cells indicating that they are virus-induced, nor were they present in cells still early during infection (<30 hpi) before VP1 expression (data not shown).

### Electron Tomography of “Virus Factories”

**Figure 2:**
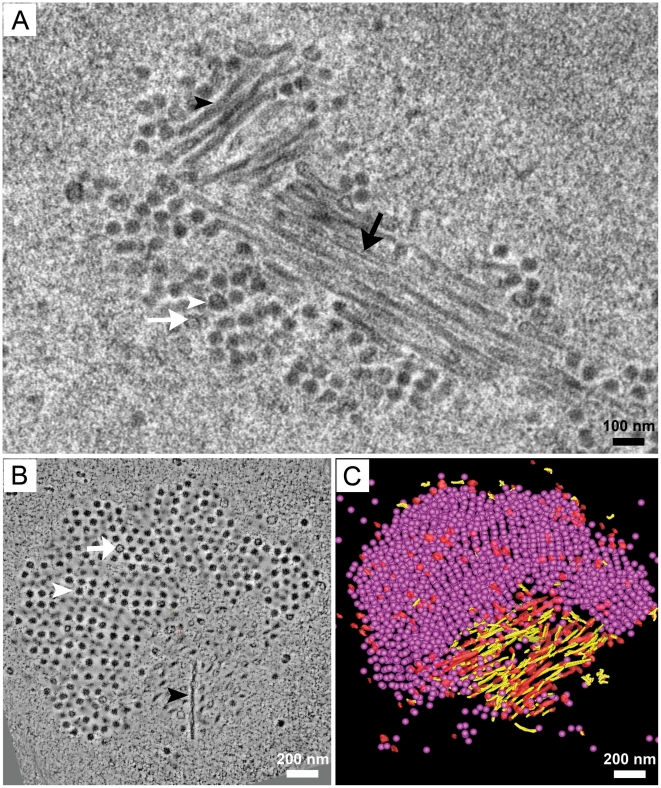
Tomographic reconstruction of a virus factory. 3T3 cells were infected with PyV as described for Figure 1. **A**) 70 nm thin section of Epon-embedded cells showing tubular structures adjacent to a virus cluster. (Black arrowhead, “full” tubular structure; white arrowhead, full virion; black arrow, “empty” tubular structure; white arrow, empty virion) **B**) A cluster of virions packed in a highly ordered array at the periphery of a bundle of tubular structures. One of the tubular structures appears in the plane (black arrowhead). Empty (white arrow) and full (white arrowhead) virions are identified in the virus clusters. **C**) 3-D model of (B) showing ≈2000 full assembled virions (pink spheres) and ≈2% empty virions (red spheres) in a 300 nm thick section. The tubular structures are either filled with electron-dense material (yellow cylinders) or appear empty (red cylinders). (See also Supporting information Video S2).

The diameter of the virions in these preparations was approximately 40 nm, which is consistent with shrinkage due to sample preparation. When the tubular structures were imaged in thin-sections (70 nm) of plastic-embedded PyV-infected 3T3 cells, their average diameter was 35–40 nm, and two types could be distinguished based upon the presence or absence of an electron dense core (Figure 2A). This distinction is similar to the differences in electron densities seen for empty *versus* full virus capsids (Figure 2A), where full capsids display a dark, electron dense core due to the presence of DNA (Figure 2A).

In order to obtain a 3-D representation of the virus factories, a semi-thick (300 nm) section was imaged as a dual-axis tilt-series (±60° in 1° increments, 200 kV) in the electron microscope. The tomographic reconstructions of a single virus factory revealed that the virus cluster contained predominantly full virions, and the tubular structures exhibited a “full” and “empty” morphology similar to what was observed in thin sections and for virions (Figure 2B). 3-D modeling of the virus factory revealed that the majority of the tubes were “full” and that their orientation varied with respect to the virus cluster (Figure 2C; see Supporting information Video S2).

### Ultrastructure of a Virus Factory

**Figure 3:**
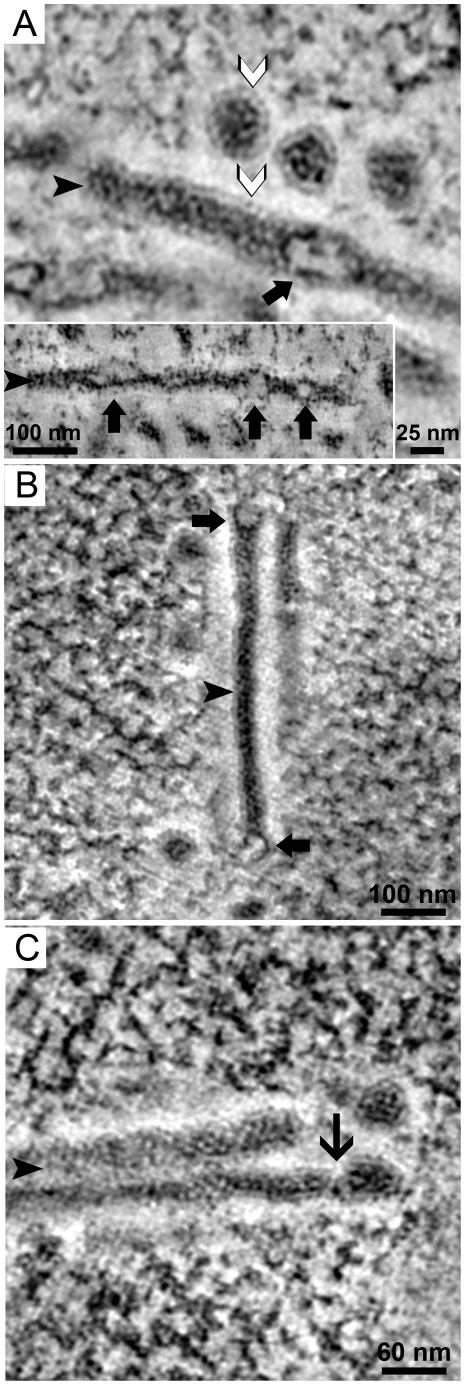
Tubule ultrastructure and association with virions. PyV-infected 3T3 cells (see Figure 1) were processed by high pressure freezing and cryo-substitution for electron microscopy and a series of images was extracted from dual-axis tomograms of Epon-embedded, 300 nm thick sections. **A**) Two distinct virions show a lighter and well-arranged density at their periphery (white arrowhead) corresponding to capsid protein density as well as a dense core suggesting DNA. The tubular structure exhibited a similar organization with a lighter and well-arranged density at its periphery (white arrowhead) as well as a patched and electron-dense core (black arrowhead). Occasionally, “bulges” or opaque regions (thick black arrows, also see inset) are seen along the length of the tube. **B**) A tubular structure (black arrowhead) exhibiting bulges forming at both ends (thick black arrows). The bulges are defined by an inner layer of dense material surrounded by well-arranged lighter densities. **C**) A mature virion is partially attached to the tubular structure (black arrowhead). A constriction point is seen where the virion may be shed from the tubular structure (thin black arrow). (See also Supporting information Figure S1).

At high resolution, the virions appeared to have a dense core, representing the viral genome, surrounded by a lower density region. This outer density was consistent with the thickness of the capsid shell. The tubular structures had a remarkable substructure. In cryo-substituted, Epon-embedded preparations the tubes appeared to have a dense core surrounded by a lighter, uniform density similar to that seen for virions (Figure 3A). Along the tubes “bubble-like” structures were observed (Figure 3A inset) similar to that observed in the tomogram of the single virus factory (see Supporting information Video S2). The ends of the tubes also had bubble-like structures (Figure 3B), but were sometimes seen as blunt (Figure 3C). Occasionally, the end of a tube was attached to a virion, with a constriction that suggested a detachment point of a nascent viral particle (Figure 3C).

**Figure 4:**
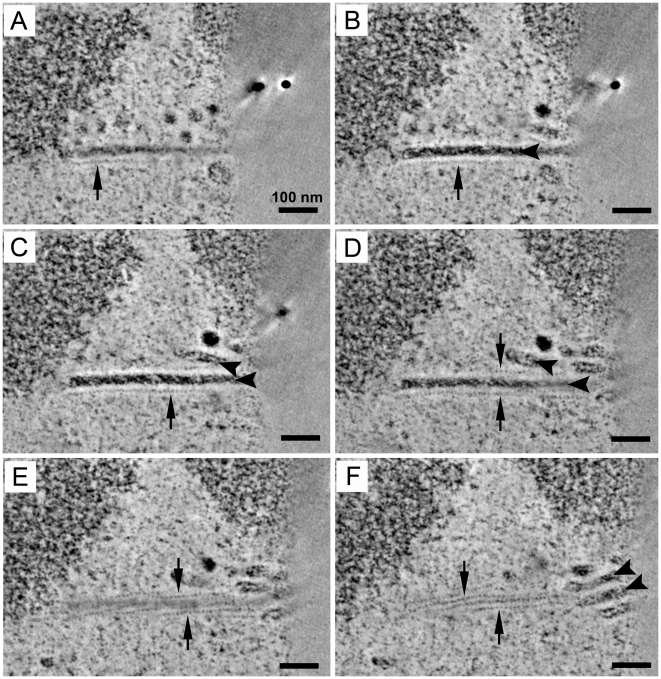
Periodic densities are associated with the tubes. Images from a dual-axis, tomographic reconstruction of PyV-infected 3T3 cells (described in Figure 1) extracted *in silico* every 5 nm. **A–F**) Sections (5 nm) were extracted from the tomogram every 6 nm in the z-plane allowing a longitudinal view of a tubular structure (black arrowhead) and its associated periodical filamentous structures (black arrow). The electron-dense material within the tubular structure has a diameter of 33 nm. The fine, periodic structures were 10 nm from the core with a periodicity of 8–12 nm.

Further ultrastructural information was obtained from tomographic reconstructions in which *in silico* 5 nm thick slices were extracted every 6 nm to yield a detailed examination of the 3-D volume comprising the tubes and their periphery (Figure 4A–F). Tomographic analysis again revealed a dense core of the tube surrounded by a well-defined rim of lower density, similar to that described above for both tubes and virions (Figure 3). Further structural analysis of the low density rim of the tube revealed that its surface was marked by periodically arranged high electron densities (8–12 nm apart). The density of these dots appeared to follow a helical path around the dense core (Fig. 4F). It is unclear whether the periodic structures represent viral or host proteins. The similarities between the ultrastructure of the tubular structures and progeny virions (Figures 3 and 4) support the idea that the tubes may represent an assembly intermediate during virus production.

### Association of PyV Proteins with Nuclear Structures During Infection

**Figure 5:**
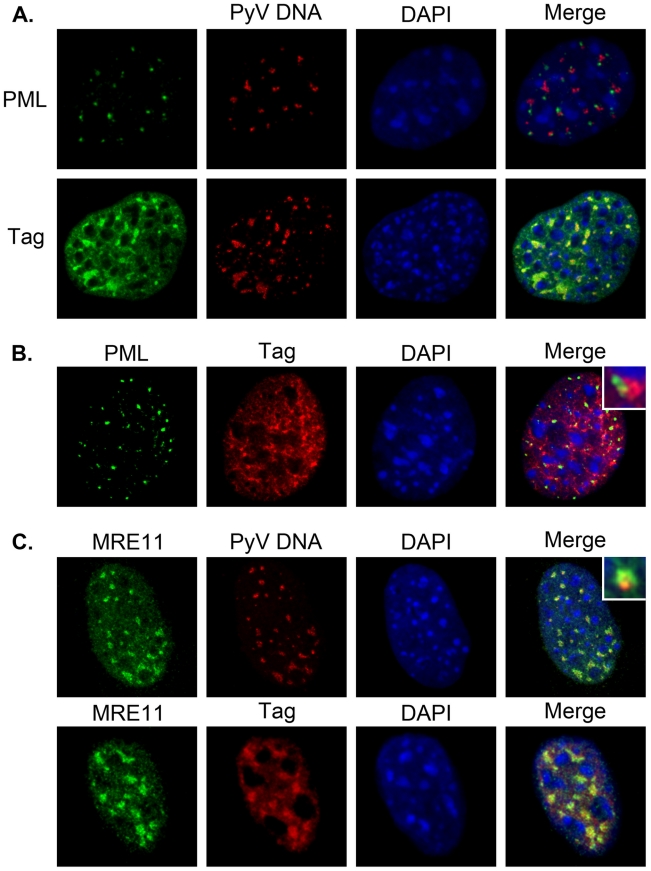
PyV DNA and T-antigen are localized near PML-NBs. C57 MEFs were infected at an MOI of 30–40 pfu/cell. At 24 or 28 hpi cells were fixed, permeabilized, and co-stained with either anti-PML, anti-Tag and/or anti-MRE11 antibodies followed by AlexaFluor-conjugated secondary antibodies, a fluorescently-labeled PyV DNA FISH probe, and DAPI staining of nuclei. **A**) PyV-infected C57 MEFs at 24 hpi stained for either PML (top) or Tag (bottom) followed by fluorescent *in situ* hybridization (FISH) for PyV DNA. **B**) PyV-infected C57 MEFs at 28 hpi co-stained for PML and Tag. **C**) C57 MEFs at 24 hpi co-stained for either MRE11 and PyV DNA (by FISH) or MRE11 and Tag. All images represent a 0.1 mm z-stack slice. Insets show enlarged regions from image to illustrate the localization of proteins in relation to each other (B) or PyV DNA (C).

SV40, BKV and JCV viral DNA and proteins have been previously localized to PML-NBs by immunohistochemistry [17], [28]–[30], and we determined if PyV DNA and proteins were similarly localized. PML-NBs were identified using antibodies directed against the PML protein. Viral proteins were visualized by staining PyV-infected C57 mouse embryo fibroblasts (MEFs) for Tag at 22–28 hpi. Similar results were obtained when PyV-infected 3T3 fibroblasts were used (data not shown). Using a fluorescently-labeled (FISH) probe specific for viral DNA, we found PyV DNA localized adjacent to PML-NBs in C57 MEFs at 24 hpi (Figure 5A, top panel). Tag was localized diffusely throughout the nucleus, with a subset of staining in more intense puncta co-localizing with PyV DNA (Figure 5A, bottom panel). At later times (28 hpi), the intensity of Tag staining in the nucleus increased while its localization pattern remained similar to that seen earlier during infection (compare Figure 5A to Figure 5B). When co-stained for PML-NBs and Tag at 28 hpi, we found that Tag localized around the PML-NBs (Figure 5B). At 28 hpi, PML-NBs appeared larger in size when compared to uninfected cells, consistent with previous observations for BKV and SV40 [30], [32]. At either time, imaging through multiple z-planes revealed that the Tag staining surrounded the PML staining while the PyV DNA stained areas adjacent to PML. This pattern of localization is similar to that previously observed for SV40 Tag during SV40 infection [32].

PML-NBs have been associated with host proteins responsible for transcriptional regulation, apoptosis, and DNA damage repair. The DNA repair complex, MRN, is comprised of three interacting proteins, MRE11, Rad50 and Nbs1. MRN is recruited to sites of double-strand DNA (dsDNA) breaks, including those organized at or near PML-NBs [41], [42] and MRE11 is recruited to SV40 sites of replication but later degraded [32]. Using a combination of immunohistochemistry and FISH, we determined whether MRE11 localized with PyV DNA during infection. In uninfected cells, the MRE11 staining was faint, presumably due to its diffuse localization throughout the nucleus (data not shown). Upon infection, MRE11 staining was increased and became localized in discrete nuclear foci around the viral DNA (Figure 5C, top panel). When co-stained for Tag and MRE11, the MRE11 foci co-localized with the bright Tag puncta (Figure 5C, bottom panel). These data indicate that PyV also may recruit MRE11 to sites of viral DNA replication.

### Proteins Associated with the Factories

**Figure 6:**
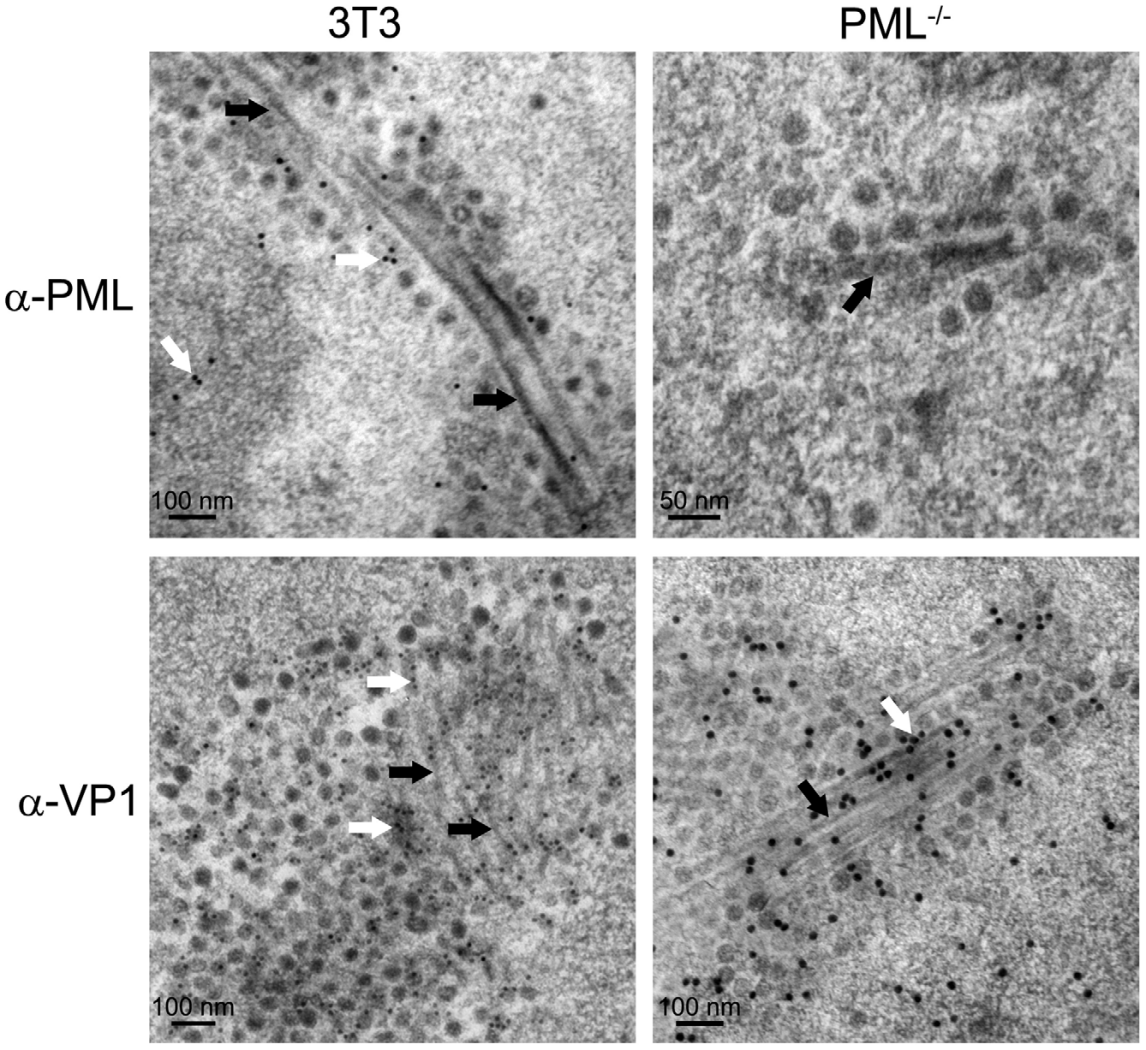
Tubular structures contain VP1. PyV-infected 3T3 cells or PML^−/−^ MEFs (MOI of 10–20 pfu/cell) at 32 hpi were frozen by high pressure and processed by cryo-substitution for immunoelectron microscopy. Thin sections (45 nm or 70 nm) of Lowicryl-embedded samples were stained with either anti-PML or anti-VP1 antibodies followed by a secondary antibody conjugated to 10 or 15 nm colloidal gold. Top panels: 45 nm sections stained for the PML protein; white arrows, anti-PML staining, black arrow, tubular structures. Bottom panels: 70 nm sections stained for VP1; white arrows, anti-VP1 staining, black arrows, tubular structures.

To identify protein components of the virus factory, thin sections of virus-infected cells were stained with antibodies against VP1 and PML, using immunogold electron microscopy. The anti-PML staining was observed throughout the nucleus and occasionally within the virion clusters and near the tubes, but was not specifically associated with the tubular structures (Figure 6). The tubular structures of the virus factory stained positively for VP1 (Figure 6). These data indicate that the tubular structures are comprised of VP1, although the presence of other host proteins cannot be excluded.

### PML is Not Required for Virus Factory Formation

**Figure 7:**
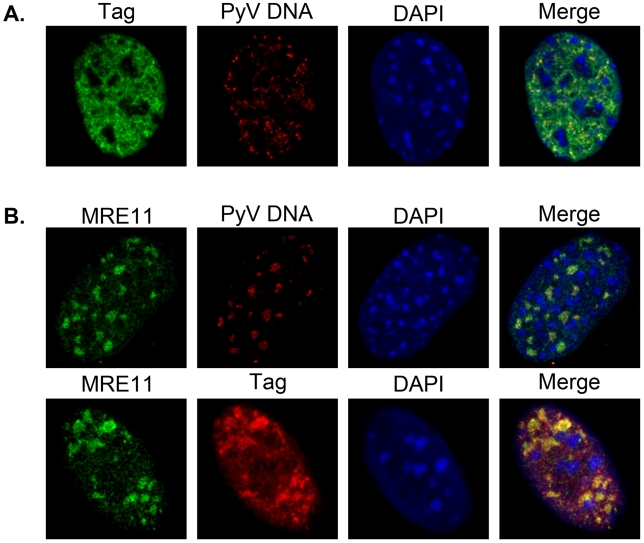
PyV DNA and T-antigen localization in PyV-infected PML^−/−^ MEFs. PML^−/−^ MEFs were infected with PyV at an MOI of 30–40 pfu/cell. At 22 or 24 hpi cells were fixed, permeabilized, and co-stained with either anti-Tag and/or anti-MRE11a antibodies followed by AlexaFluor-conjugated secondary antibodies, a fluorescently-labeled PyV DNA FISH probe, and DAPI staining of nuclei. **A**) FISH for PyV DNA at 24 hpi followed by antibody staining for Tag. **B**) Infected cells were stained by FISH for PyV DNA at 22 hpi followed by antibody staining for MRE11 or co-stained for MRE11 and Tag. All images represent a 0.1 mm z-stack slice.

The PML protein has been suggested to function as a scaffold for other PML-NB-associated proteins. When PML expression is disrupted, the PML-NB-associated proteins (*i.e.*, Sp100, Daxx, SUMO-1) do not localize in nuclear dots when analyzed by immunofluorescence staining [43]. Since we observed that PyV DNA and Tag localized around PML-NBs, we determined their localization in MEFs isolated from PML-knockout mice (PML^−/−^ MEFs) [44]. In PyV-infected PML^−/−^ MEFs, PyV DNA was localized in small, punctate patches (Figure 7A), similar to its localization in C57 MEFs and 3T3 fibroblasts (Figure 5A; data not shown). In addition, Tag and MRE11 localization in infected PML^−/−^ MEFs was similar to that observed in wild-type infected fibroblasts (Figure 7B). Tubular structures were also observed in close proximity to virion clusters in these cells, similar to those seen in PyV-infected 3T3 cells and C57 MEFs (Figure 6; data not shown). Immunogold labeling of PML^−/−^ MEFs revealed that VP1 stained the virus factories similarly to the factories found in 3T3 cells (Figure 6). Together these data suggest that the PML protein is not necessary for the punctate localization of PyV DNA, its associated proteins, or the formation of virus factories.

### PML is Not Required for Virus Replication *in vitro* or *in vivo*

The observation that virus factories were present in PyV-infected PML^−/−^ MEFs suggested that PyV replication is not impaired in these cells. To test whether PML^−/−^ MEFs assembled and released virus, viral DNA (Hirt DNA, [45]) and supernatants from infected cells were isolated at various times after infection. We found that viral DNA accumulation was similar between 3T3 cells, C57 and PML^−/−^ MEFs (data not shown). At 3 and 4 days post-infection (dpi), supernatants from cells were analyzed for the presence of packaged viral genomes. Supernatants were digested with DNase to remove unprotected DNA prior to proteinase K digestion, and then analyzed by qPCR for viral DNA. The supernatants from 3T3 cells, C57 and PML^−/−^ MEFs contained similar amounts of virus particles by this assay (Table 1). Furthermore, these supernatants contained similar titers of infectious virus, as determined by plaque assay (Table 1). Therefore, PML is not required for PyV replication, assembly or release of infectious virus *in vitro*.

**Table 1:** The PML Protein is not required for virus replication in vitro.

	Three days post-infection		Four days post-infection	
Supernatants^a^	NRG^b^ per ml	PFU^c^ per ml	NRG per ml	PFU per ml
3T3	2.5×10^10^±1.8×10^9^	3.3×10^6^	2.7×10^10^±8.0×10^8^	3.6×10^6^
C57	2.6×10^10^±1.4×10^9^	2.3×10^6^	4.1×10^10^±3.2×10^9^	1.2×10^6^
PML^−/−^	1.4×10^10^±4.8×10^8^	2.0×10^6^	1.5×10^10^±5.1×10^8^	8.0×10^6^

a Indicated by the name of the cell type from which the supernatant was harvested after infection with PyV.

b NRG – nuclease-resistant genomes as determined by qPCR.

c PFU – plaque-forming units.

The *in vivo* progression of PyV infection was compared between PML^−/−^ mice [44] and the syngeneic mouse strain (Sv129) using qPCR for viral DNA in isolated tissues. Sv129 or PML^−/−^ mice were infected intraperitoneally with mouse polyomavirus strain A2 and mice were sacrificed 6 or 14 days later. Total DNA was isolated from homogenized tissue and PyV genomes were quantitated by qPCR (Table 2). The level of viral genome replication was similar between wild-type and PML^−/−^ mice on days 4 and 6 (Table 2; data not shown). On day 14, however, although the kidneys had very similar PyV load in PML^−/−^ mice and the wild type controls, the liver and lung tissues of PML^−/−^ mice had lower viral genome levels than wild type mice. By day 14, the adaptive immune response starts to control the infection, therefore data from mice infected for 6 days provided a more reliable readout of the ability of the virus to replicate *in vivo*.

**Table 2:** The PML protein is not required for virus replication *in vivo*.

	Six days post-infection^a^		Fourteen days post-infection^a^	
Mouse Tissue	Wild-Type	PML^−/−^	Wild-Type	PML^−/−^
Spleen	2.8×10^6^	2.7×10^6^	5.1×10^5^	8.0×10^4^
Liver	1.1×10^6^	2.6×10^6^	1.1×10^6^	1.2×10^5^
Kidney	3.0×10^6^	5.0×10^6^	1.2×10^5^	1.2×10^5^
Lung	2.3×10^5^	3.2×10^5^	2.4×10^5^	1.0×10^4^

a Mean values shown as PyV genome copies/µg tissue DNA.

## Discussion

Many viruses appear to assemble progeny virions at specific intracellular locations, often termed “virus factories.” These sites may represent subcellular scaffolds where replicated genomes and capsid proteins spatially intersect in an efficient and coordinated manner to assemble virions. We used electron microscopy and tomographic 3-D reconstructions to identify possible sites and structural features for PyV virus assembly. We identified tubular structures in close physical association with progeny virions in the nucleus of PyV-infected mouse fibroblasts. The chronology of tube appearance suggests that these structures form at the initiation of virion assembly. Tubular structures were not observed in the cytoplasm of either infected or uninfected cells. We have termed the tubular structures, together with the associated virion clusters, virus factories.

The tubular structures appear similar to “filamentous” structures previously observed in samples prepared by conventional, chemical fixation techniques and visualized by electron microscopy in PyV-infected baby hamster kidneys [46] and secondary mouse embryo cell cultures [47], COS-7 cells transfected with expression plasmids encoding JCV viral proteins 29,48, JCV-infected brain tissue [40], [49], [50], and tissue samples from PML patients [51], [52]. Consistent with the findings in these previous reports, we observed the accumulation of tubular structures early after infection and their relative absence late in infection when the nucleus was filled with virions [47]. In these early studies the tubular structures were also suggested to be assembly intermediates [46], [47], [53], [54]. Tubular structures also have been reported in studies of papillomavirus isolated from human and rabbit warts [55], and of PyV isolated from infected tissue culture cells [56], [57]. Although we have not yet identified similar structures in BKV or SV40-infected cultured cells (preliminary findings), the observation of tubular structures in histologic sections from PyV-infected mouse or hamster kidneys, PML patients, and human papillomavirus specimens supports their physiologic relevance to infection.

Previous reports modeled two types of tubes from negatively-stained and low dose electron microscopic images. The tube types, termed hexamer or pentamer, are distinguished by the arrangement of VP1 pentamers within the tubes [56], [57]. The hexamer tubes have an average diameter of 45–55 nm whereas the pentamer tubes have a diameter of ≈30 nm [55], [56]. Thus, the tubular structures we observed during infection may represent the hexamer or pentamer tubes. We observed tubes with varying diameters (30–45 nm), which is consistent with the different diameters of the isolated tubes. The variation in diameter may be due to fixation and preservation of the samples isolated under various conditions. In contrast, however, the tubes we observed often had an electron dense core. In addition surrounding the electron-dense core (Figure 4) we could identify a less dense rim associated with periodic densities (“dots”) spaced ≈8–12 nm apart. This distance is consistent with the distance between the centers of each pentamer in a hexamer tube [55]–[57]. Furthermore, the helical twist of pentameric subunits in pentamer/hexamer tubes is consistent with the apparent twisting of the filamentous structure around the tube core. Although the filamentous structures surrounding the tubes likely are comprised primarily of VP1 pentamers, it is possible that host proteins are also associated with the structures.

The defining step in encapsidating large DNA viruses has been envisioned as the injection of the viral genome into a preformed capsid structure, similar to phage assembly. The current view of small DNA virus encapsidation is vague, hypothesizing that coat proteins are polymerized around the viral minichromosome in some manner. Our images suggest an alternative view. In comparison with virions in the same sections, the density surrounding the tubes corresponded to the density of the outer capsid of the virions, while the dense core was similar to the virion interior. Along with the occasional appearance of “budding” virions from the ends of the tubes, it might be envisioned that virion encapsidation proceeds in a manner akin to making sausage. The VP1 pentamers would first polymerize into tubes, and the viral chromatin then traverses the interior of the tubes until a terminal sealing event buds off the icosahedral particle. This model has an obvious relation to the assembly of large DNA viruses in that a preformed structure is subsequently filled with the viral genome. We present a model of icosahedral virus budding from the tubes in supporting information Figure S1.

Although PML-NBs have been previously speculated to be sites of viral DNA replication and possibly virion assembly, we found that the loss of the PML protein itself did not affect the punctate localization of PyV Tag or DNA (Figure 7), or the presence of tubular structures (Figure 6). Thus, while the nuclear bodies as defined by PML immunohistochemistry are not required for virus assembly, it is possible that other components of the PML-NBs may remain in a focal distribution to facilitate viral DNA replication, packaging and virion assembly. In particular the continued localization of MRE11 in puncta in the absence of PML (Figure 7) suggests that important functions continue in discrete foci. As yet we have not been able to directly associate the virus factories we observe in the EM sections with replicating viral DNA, Tag, or PML seen by immunohistochemistry. Although the virion clusters appear in discrete patches and could represent the foci of viral DNA seen by FISH, the currently available antibodies for PML, MRE11 and Tag, have not performed well in the freeze-substituted sections for immunoEM, presumably because the epitopes were destroyed during sample preparation. However, the immunohistochemistry consistently localizes PyV DNA and Tag around the PML staining, suggesting that replication may be occurring adjacent to but not directly at the site of the factories.

PML-NBs have been suggested to be part of an intrinsic cell defense against the replication of a variety of viruses [22]. Both PML and Sp100 are interferon-inducible genes [58], [59], and their protein levels correlate with the increased size and number of PML-NBs after IFN induction [60]–[63]. Large DNA viruses such as HSV-1 and Ad5 specifically target PML-NB structures during their replication cycle. PML-NBs are modified by the HSV-1 ICP0 protein, which induces proteasome degradation of the PML protein [64]–[66] whereas the Ad E4 ORF3 protein disrupts PML-NBs by reorganizing PML into fibrous strands [19], [67]–[69]. Without these viral proteins, viral replication is reduced for both HSV-1 and Ad. Likewise, certain RNA viruses obtain a replication advantage when PML expression is disrupted. For example, when PML^−/−^ mice were infected with either vesicular stomatitis virus (VSV) or lymphocytic choriomeningitis virus (LCMV), an increase in virus production was observed [60]. However, PML-NBs do not serve as a site of assembly for these RNA viruses, and the anti-viral effects may be a consequence of a separate function of PML-NBs. Our findings represent the first study which demonstrates both *in vitro* and *in vivo*, that loss of PML expression does not affect virus replication. These results are consistent with data from other polyomaviruses (BKV, JCV) using cells with shRNA knockdown for PML or arsenite-treatment to disrupt PML protein expression [30], [70]. Unlike JCV, however, we did not see an enhancement of virus production when PML was absent [70]. Furthermore, we did not see a rearrangement of PML-NBs such as observed during BKV infection [30], although a slight increase in the size of PML-NBs during infection was noted.

The structural features of virion assembly factories are beginning to be defined for many viruses. Viruses frequently yield important insights for cell biology, and the structures facilitating virus assembly may also inform hypotheses concerning the organization of important nuclear functions such as for DNA repair. The multiple functions assigned to PML-NBs are undergoing continued revision with respect to host-virus interactions. The role of PML-NBs in PyV replication is complex, and PML-NB associated proteins other than PML itself may provide the necessary architectural foundation for the tubular structures that appear to be the PyV factories.

## Materials and Methods

### Ethics Statement

This study was carried out in strict accordance with the recommendations in the Guide for the Care of Use of Laboratory Animals of the National Institutes of Health. The protocol was approved by the Institutional Animal Care and Use Committee of the University of Massachusetts Medical School (Approval number: 305), following recommendations by the Office of Laboratory Animal Welfare (OLAW). All mouse strains were maintained in specific pathogen–free conditions in the animal facilities of the University of Massachusetts Medical School. All efforts were made to minimize suffering and provide humane treatment to the animals included in the study.

### Cells and Viruses

A31 3T3 fibroblasts were grown in DMEM (D6429; Sigma) supplemented with 10% bovine calf serum (BCS) and penicillin/streptomycin. C57 mouse embryonic fibroblasts (MEF) were obtained from ATCC (SCRC-1008; Manassas, VA) and served as a wild-type MEF control. PML^−/−^ MEFs were a gift of Gerd Maul [43], [44]. Both MEFs were grown in DMEM supplemented with 20% fetal bovine calf serum (FBS) and antibiotics. Virus strain NG59RA [71] was used for all cell culture infections and strain A2 [72] for mouse infections. Cell culture infections were carried out as described previously [73]. Cells were infected at a multiplicity of infection of 10–20 pfu/cell for 32–34 hours for electron microscopy and replication studies, and 30–40 pfu/cell for 22–24 hours for immunofluorescence analysis. A higher MOI was used for immunofluorescence studies to compensate for cell loss during the staining process. Similar results were observed when an MOI of 10–20 pfu/cell was used although it was more difficult to identify infected cells due to loss during staining. Similar results were observed at 32–34 hours post-infection (hpi) when cells were stained for immunofluorescence analysis. However, cell loss was significantly increased at later times and the fluorescence signal was saturated. Thus, to define protein localization, we chose to show earlier times after infection for the immunofluorescence studies.

### Immunofluorescence

T-antigen was detected using rat anti-T-antigen (E3, gift of Tom Benjamin). PML was detected using mouse anti-PML (ALX-804-816; Enzo, Plymouth Meeting, PA) and MRE11 was detected using rabbit anti-MRE11a (B1447; LSBio,). Primary antibody dilutions for rat anti-Tag, mouse anti-PML, and rabbit anti-MRE11a were 1∶5000, 1∶1000 and 1∶1000, respectively. Secondary antibodies were either AlexaFluor 488- or AlexaFluor 594-conjugated (Invitrogen) and were diluted 1∶1000. All antibodies were diluted in 10% FBS/PBS.

Cells were grown on acid-etched, poly-L-lysine-coated coverslips and infected with NG59RA as described above except cells were harvested at 22–24 hpi. Cells were prepared for immunofluorescence staining according to Zhao *et al* [32], with some modifications. Briefly, cells were washed 3 times with cold phosphate-buffered saline (PBS) solution followed by cytoskeleton buffer (CSB) [10 mM piperazine-*N*,*N*-bis(2-ethanesulfonic acid) (PIPES), pH 6.8, 100 mM NaCl, 300 mM sucrose, 1 mM MgCl_2_, 1 mM EGTA]. Soluble proteins were pre-extracted for 3 min with cold CSB containing 0.5% Triton X-100 and protease inhibitors (Complete mini tablets, Pierce) at 4°C. The cells were then washed with PBS, fixed in 4% paraformaldehyde (PFA) in PBS for 30 min, washed with PBS, and blocked with 10% FBS/PBS at 4°C. Samples were incubated with Image-iT FX signal enhancer (I3693; Invitrogen) for 20 min followed by incubation with primary antibodies for 1 hr at room temperature, washed with PBS, and incubated with secondary antibodies for 1 hr at 22°C. Stained cells were mounted onto glass slides with ProLong anti-fade reagent containing Dapi (P36935; Invitrogen) and allowed to incubate at 22°C overnight.

### Fluorescence *in situ* Hybridization

To detect Py viral genomes after infection, we used nick-translation to label DNA probes specific for PyV DNA. Briefly, the entire Py viral genome (NG59RA) was cloned into pUC18 at BamHI (pUC-PyV) and 2 µg plasmid DNA was labeled with SpectrumRed using the Vysis Nick Translation Kit (Abbott Molecular, Des Plaines, IL), according to the manufacturer protocol. Labeled DNA was ethanol-precipitated with herring sperm and human Cot-1 DNA and resuspended in 20 µl cDen-Hyb (Insitus Biotechnologies, Albuquerque, NM) to yield a final probe concentration of 100 ng/µl.

FISH analysis was performed as described previously [28], with some modifications. Briefly, cells grown on coverslips were infected, fixed and immunostained for viral or host proteins, as described above. Immunostained cells were fixed a second time with 4% PFA in PBS to crosslink bound antibodies followed by treatment with 0.2 mg/ml RNase Type III (Sigma) in 2X SSC at 37°C for 15 min and washed in 2X SSC 3 times. The PyV DNA probe was diluted in cDenHyb and hybridized to samples for 2 min each at 80°C, 70°C, 60°C, 50°C and 45°C followed by an overnight incubation at 37°C. Coverslips were washed at 45°C with 1.5X SSC, 50% formamide/1.5X SSC, and 1.5X SSC for 5 min each. Stained cells were mounted onto glass slides with ProLong anti-fade reagent containing Dapi (Invitrogen) and allowed to incubate at 22°C overnight.

### Confocal Microscopy

Imaging of fixed cells was performed with an inverted fluorescence microscope (TE2000-U; Nikon) equipped with an electron-multiplying charge-coupled device camera (Cascade II; Photometrics) and a Yokogawa spinning disc confocal system (CSU-Xm2; Nikon). Images were taken with a 60X NA 1.4 oil objective using MetaMorph (version 7.0; MDS Analytical Technologies) software. The data from each channel (excitation wavelengths at 488, 543 and 633 nm) were collected sequentially using the appropriate band-pass filters built into the instrument. For z-stacks, optical slices were taken at 0.2 µm increments. Data sets were processed using ImageJ (National Institutes of Health) software. Data are shown as an extracted slice from the z-stacks.

### High-Pressure Freezing

Infected cells were trypsinized and collected by centrifugation. The cell pellet was resuspended in growth media supplemented with either 20% dextran (40,210 Da, Sigma–Aldrich) or 150 mM mannitol (M9647; Sigma). For dextran-prepared samples, 3 µl of the cell suspension was deposited into an aluminum planchette (Engineering Office M. Wohlwend GmbH, Sennwald, Switzerland) with a sample depth of 100 µm and vitrified in the BalTec HPM 010 under a pressure of 2000 bar by a jet of liquid nitrogen applied on the carrier.

For mannitol-prepared samples, cells were collected by centrifugation, the supernatant was poured off and a slurry was made by resuspending the cell pellet in the remaining supernatant. 1–5 µl of the cell slurry were transferred to an aluminum planchette and frozen as described above. The frozen cell suspensions were stored in liquid nitrogen prior to cryo-substitution and plastic embedding.

### Cryosubstitution

Dextran-prepared samples: High-pressure frozen cells were cryo-substituted with 1% osmium tetroxide/0.2% uranyl acetate/acetone over three days at −90°C with a gradual warming to room temperature over a period of 2 days. Samples were removed from planchettes, and rinsed with acetone several times to remove residual osmium and uranyl acetate. Cells were infiltrated with increasing concentrations of Epon-Araldite 802 epoxy resin (Electron Microscopy Sciences, Port Washington, PA) over a period of 2 days, with 3 final incubations of 100% Epon to remove residual acetone. Samples were polymerized in BEEM embedding capsules (Electron Microscopy Sciences) by addition of DMP-30 accelerator and incubation at 60°C for 2 days.

Mannitol-prepared samples: High-pressure frozen cells were cryo-substituted and prepared as described above for Epon epoxy resin embedding. For samples to be used for immunogold-labeling, frozen cells were cryosubstituted with 0.1% uranyl acetate/0.25% glutaraldehyde/acetone at −75°C for 3 days followed by a gradual warming to −35°C over 12 hr. Cells were removed from the planchettes and rinsed with −35°C acetone and infiltrated over 3 days with increasing concentrations of Lowicryl/HM20 resin (Electron Microscopy Sciences), with 3 final changes of 100% HM20. Resin-infiltrated cells were polymerized in BEEM capsules at −35°C under ultraviolet light for 2 days. Samples and resin were maintained at −35°C during infiltration steps in an automated freezing system (AFS; Leica Microsystems).

### Electron Microscopy

Epon-embedded sections were cut to a thickness of 70 nm with an UltraCut–UCT microtome (Leica Microsystems) using a diamond knife (Diatome, Biel, Switzerland). Sections were picked up on Formvar-coated copper slot grids and stained with 2% aqueous uranyl acetate for 10 min, followed by Reynold's lead citrate [74] for 4 min. Images were obtained on a Philips CM100 electron microscope operating at 80 kV.

### Immunogold Electron Microscopy

For immunogold labeling, Lowicryl-embedded samples were sectioned at a thickness of 45 nm (anti-PML) or 70 nm (anti-VP1) and picked up on Formvar-coated, copper slot grids. Sections were fixed with 0.5% paraformaldehyde (diluted from a fresh stock (32%, Electron Microscopy Sciences) in PBS) for 15 min at 22°C. The grids were rinsed with PBS and blocked in 1% milk/PBST* (PBS/0.02% Tween 20) in a humidified chamber for 30 min at room temperature followed by incubation in the same chamber on droplets of primary antibody diluted in 1% milk/PBST* for 2 hr at 22°C. The grids were washed in a stream of PBS, blotted to remove excess PBS and incubated with gold-conjugated secondary antibody for 1 hr at 22°C. Samples were washed in a stream of PBS followed by a distilled water wash, air-dried and post-stained as described above except incubation times were reduced to 4 min and 2 min for methanolic uranyl acetate and lead citrate, respectively. Samples were imaged as described above.

Primary antibody dilutions for rabbit anti-VP1 (I58) and rabbit anti-PML (ab67761; AbCam, Cambridge, MA) were 1∶2000 and 1∶80, respectively. Secondary antibodies were 10 or 15 nm gold-conjugated goat anti-rabbit IgG (Ted Pella Inc., Redding, CA) and were diluted 1∶20. All antibodies were diluted in 1% milk/PBST*.

### Electron Tomography

Semi-thick Epon-embedded sections (∼300 nm) were stained with 2% uranyl acetate as described above for thin Epon-embedded sections. Colloidal gold particles (15 nm; BBI Research, Inc., Madison, WI) were place on both grid surfaces to serve as fiducial markers for subsequent image alignment. Sections were imaged on a FEI Tecnai F20 microscope (FEI Company Ltd., Eindhoven, The Netherlands) operating at 200 kV and images were collected on a 4K by 4K CCD Ultrascan camera (Gatan, Inc., Pleasanton, CA). Dual-axis tilt series data sets were acquired using the SerialEM software package [75]. 1×1, 2×1 or 2×2 montages were acquired on either a single section or up to six serial sections (Figure 1D and Supporting information Video S1). The nominal defocus was set to 0.5 µm and the pixel size of the data varied between 0.764 and 1.206 depending on the magnification used. Tomographic reconstructions were produced by the IMOD software package [76]. 3-D structures of interest were surface rendered using tools available in the IMOD software package (Figures 1D and 2C and videos).

### Viral Release Assay

10^5^ cells per 60mm dish were seeded 12–16 hrs before infection with NG59RA. At this density, the cells were 50–60% confluent at the time of infection. Virus preparation and infection was carried out as described above. At times indicated, supernatants were transferred to 15 ml conical tubes and saved. Cells remaining on the plate were treated with neuraminidase (NA) Type V (Sigma) diluted in NA buffer (10 mM Hepes, pH 5.6/1 mM CaCl_2_/1 mM MgCl_2_/5 mM KCl) at 37°C for 30 min. The NA supernatant was collected and combined with the cell supernatant. The plates were washed with PBS 3 times and each wash was collected and combined with supernatants. The combined supernatants and washes were stored at −20°C and are referred to as the “viral supernatant.”

### Viral DNA Quantitation

Viral DNA was isolated from viral supernatants using the method described for Ad supernatants [77] with some modifications. Briefly, 50 µl viral supernatant was digested with DNase (RQ1, Promega) at 37°C for 1 hr. DNase-treated samples were incubated with Proteinase K (Fermentas) for 37°C for 1 hr. The viral DNA was purified using the Wizard DNA Clean-up System (Promega) according to the manufacturer protocol except 80% ethanol was used in place of isopropanol during the wash. The viral DNA was eluted with 50 µl prewarmed (80°C) milli-Q water and stored at −20°C until ready for use.

Primer Express 3.0 software (Applied Biosystems, Warrington, UK) was used to design probes and primers for amplification of a 67-bp region of the mouse polyomavirus genome (NCBI accession # NC_001515). PCR primers were synthesized by IDT and the TaqMan probe was synthesized by Applied Biosystems. The following primers were used: PyV VP1 forward primer, 5′TGGGAGGCAGTCTCAGTGAAA3′; PyV VP1 reverse primer, 5′TGAACCCATGCACATCTAACAGT3′; PyV VP1 probe, 5′CCGAGGTGGTGGGCTCTGGC3′. The TaqMan probe was labeled with FAM (6-carboxy-fluorescein) at the 5′ end and a quencher, TAMRA (6-carboxy-tetramethylrodamine) at the 3′ end. Optimal concentrations of probe and primer were determined using a concentration matrix, as described in Applied Biosystems protocols.

Quantitative PCR (qPCR) reactions were prepared in 96-well optical plates (Applied Biosystems) in a volume of 25 µl. Each reaction contained 200 nM TaqMan probe, 900 nM of each forward and reverse primer, 12.5 µl TaqMan Master Mix (Applied Biosystems) and 5 µl purified viral DNA or DNA standards. The intensity of fluorescence of the reporter label was normalized to the ROX Passive Reference, supplied in the master mix solution. DNA amplification was carried out using an Applied Biosystems 7500 Real-Time PCR Sequence Detection System using cycling conditions of 50°C for 2 min, 95°C for 10 min followed by 40 cycles of 95°C for 15 sec, 60°C for 1 min. For each run, duplicates of seven dilutions of the viral standard DNA (from 0.1 ng to 5×10^−5^ ng; pGEX-VP1 plasmid DNA), viral DNA samples and no template controls were simultaneously subjected to analysis. The number of genomes was determined as previously described [77] and is reported as “nuclease-resistant genomes (NRG) per ml.”

### Plaque Assay

NIH/3T3 cells were resuspended in absorption medium (DMEM/0.1% BSA) at a density of 1×10^6^ cells per ml. The virus stock was serially-diluted in absorption medium. Equal volumes of the cell suspension and viral dilution were mixed at 37°C, 5% CO_2_ for 1.5 hrs with gentle agitation every 15 minutes. Complete growth media (DMEM/5% BCS/penicillin/streptomycin) was added to the infected cell suspension and transferred to a six-well plate in triplicate (1×10^5^ cells/well). Cells were incubated at 37°C for 5 to 6 hrs to allow for cell adherence after which the growth media was gently removed and pre-warmed (42°C) overlay medium (DMEM/5% BCS/1 µM dexamethasone/penicillin/streptomycin/0.4% agarose) was added to each well and allowed to solidify. Samples were incubated at 37°C and 5% CO_2_ for 6–8 days. After incubation, the overlay agar was carefully removed and each well was washed with cold PBS. The monolayer was stained with crystal violet solution (1% crystal violet/formaldehyde) for 8 min, the stain decanted and each well washed with cold PBS. After PBS washes, the plates were inverted and allowed to dry before counting the plaques.

### Mouse Infection and Viral Genome Quantitation

PML^−/−^ mice were obtained from the NCI and 129S1/Svlmj wild-type control mice were purchased from Jackson Laboratories. Mice were infected intraperitoneally at 2 months of age with 5×10^5^ pfu per mouse with PyV strain A2. Mice were sacrificed 6 and 14 days post-infection and their organs (spleen, kidney, liver, lung) were isolated to measure viral load by qPCR.

To determine the viral load in infected mouse tissues by qPCR, DNA was prepared from organ homogenates by proteinase K (Sigma) digestion at 55°C overnight, followed by phenol extraction and RNase-A treatment (10 units/µl, Promega). The PCR amplification was performed as described previously [78]. Briefly, a 50 µl reaction mixture with 2X SYBR green master mix (4309155, Applied Biosystems) and 0.1 mM each of forward and reverse primers (Invitrogen) was prepared. The following primers were used: β actin forward, 5′CGA GGC CCA GAG CAA GAG AG3′; β actin reverse, 5′CGG TTG GCC TTA GGG TTC AG3′; PyV VP1 forward, 5′CCC CCG GTA CAG GTT CAG TCC CAT CAT3′; VP1 reverse, 5′GGC ACA ACA GCT CCA CCC GTC CTG CAG3′. The amplification for VP1 started with one cycle at 95°C for 10 min, 37 cycles of 95°C for 30 sec, 65°C for 20 sec, 72°C for 45 sec. PCR amplification with the β actin primers started with one cycle at 95°C for 10 min, then 40 cycles of 95°C for 30 sec, 62°C for 25 sec, and 72°C for 25 sec. Negative controls included a sample with no DNA template and DNA from uninfected mouse organs. Three-fold serial dilutions of DNA prepared from uninfected mouse organs (spanning 1 µg–31 ng) were used to generate a standard curve for β actin PCR. For PyV we used a recombinant plasmid containing the VP1 coding sequences and made dilution series from 2×10^8^ copies to 20 copies. All the reactions were run in duplicates. The obtained PyV copy numbers were normalized for β actin which reflected the amount of mouse genomic DNA present, and the results were expressed as PyV genome copies per µg organ DNA.

## Supporting Information

Figure S1 **Nanotube model of a tube with a budding icosahedral virus.** A model of a virus budding from a tubular structure based upon the assembly and symmetry of carbon nanotubes. Consistent with the data of Baker et al [56]–[57] the tubes are modeled as hexamers, but at the ends the symmetry necessarily includes pentamers. Similar symmetry elements are seen in HIV Fullerene capsid cones (Ganser-Pornillos, B., et al (2008) Curr Opin Struct Biol **18**:203). **A**) Tube without budding virus; **B**) Tube with budding virus; **C**) An “end-on” view of the virus budding from a tube. The budding virus has the same diameter as the tube (∼45 nm). The structures were generated using the Nanotube Modeler software from JCrystalSoft.
(TIF)

Video S1 **Tomogram and model of a PyV-infected nucleus.** A movie compiled from images obtained as a 2×2 montage over six serial thick sections (300 nm) with each section imaged as a tilt-series (±60°) of a PyV-infected 3T3 nucleus (1.8 µm total thickness). The movie progresses through the tilt-series of each section, followed by modeling of each section and concludes with a 3-D representation of the infected nucleus. Purple = condensed chromatin or chromatin-like patches; red = virions; yellow = tubular structures. Although in each section the virus factories appear separate from one other, when the sections are combined the tubular structures (yellow cylinders) appear to connect each of the virus clusters (red spheres). Single images were extracted from the movie and are shown in Figure 1B (tomogram) and 1D (3-D model).
(MP4)

Video S2 **Tomogram and model of a virus factory.** A small virus array with tubular structures adjacent to the virions. A movie was compiled from a tilt-series (±60°) of a 300 nm thick section of PyV-infected 3T3 cells. The movie proceeds through the full tilt-series followed by 3-D modeling of the virus factory. Purple = full virions, red = empty virions and empty tubes, yellow = electron-dense, or “full,” tubes. In this array both full (dark “circles”/purple spheres) and empty capsids (empty “circles”/red spheres) are seen as the movie proceeds through the tilt series. When following the tubular structures (red or yellow cylinders) through the section, “bubble-like” protrusions appear along the electron-dense, or “full,” tubes. This tomogram also reveals the close association of tubular structures with virions. Single images are shown in Figure 2B (tomogram) and 2C (3-D model).
(MP4)
