# Supplementary figures and images for: Virion Assembly Factories in the Nucleus of Polyomavirus-Infected Cells
Source: PLoS Pathog. 2012 Apr 5;8(4):e1002630. doi: 10.1371/journal.ppat.1002630 (PMC3320610; doi:10.1371/journal.ppat.1002630)

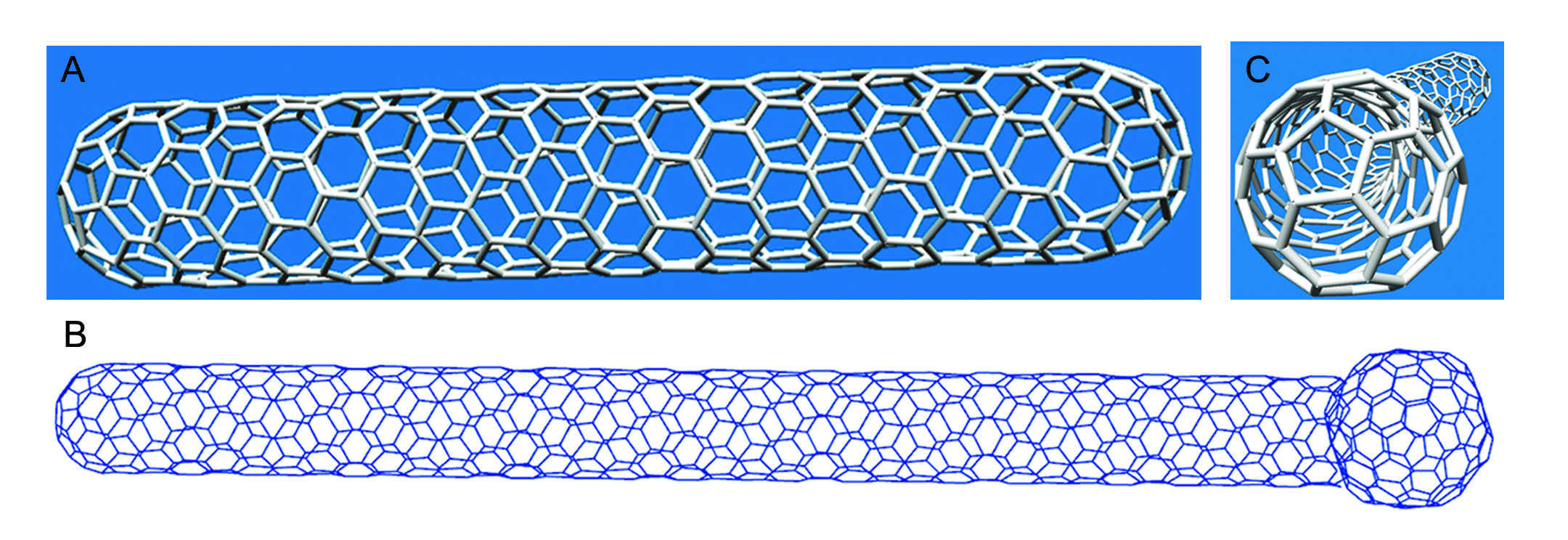

Supplement: Figure S1 — Nanotube model of a tube with a budding icosahedral virus. A model of a virus budding from a tubular structure based upon the assembly and symmetry of carbon nanotubes. Consistent with the data of Baker et al [56]–[57] the tubes are modeled as hexamers, but at the ends the symmetry necessarily includes pentamers. Similar symmetry elements are seen in HIV Fullerene capsid cones (Ganser-Pornillos, B., et al (2008) Curr Opin Struct Biol 18:203). A) Tube without budding virus; B) Tube with budding virus; C) An “end-on” view of the virus budding from a tube. The budding virus has the same diameter as the tube (∼45 nm). The structures were generated using the Nanotube Modeler software from JCrystalSoft. (TIF) [file ppat.1002630.s001.tif]
